# Detection of Porcine Circovirus 3 in Wildlife Species in Spain

**DOI:** 10.3390/pathogens9050341

**Published:** 2020-05-01

**Authors:** Ewelina Czyżewska-Dors, José I. Núñez, Viviane Saporiti, Eva Huerta, Carme Riutord, Oscar Cabezón, Joaquim Segalés, Marina Sibila

**Affiliations:** 1 Department of Swine Diseases, National Veterinary Research Institute, 24-100 Puławy, Poland; ewelina.czyzewska@piwet.pulawy.pl; 2 IRTA, Centre de Recerca en Sanitat Animal (CReSA, IRTA-UAB), Campus de la Universitat, Autònoma de Barcelona, 08193 Bellaterra, Spain; joseignacio.nunez@irta.cat (J.I.N.); viviane.saporiti@irta.cat (V.S.); eva.huerta@irta.cat (E.H.); oscar.cabezon@uab.cat (O.C.); 3 OIE Collaborating Centre for the Research and Control of Emerging and Re-emerging Swine Diseases in Europe (IRTA-CReSA), Bellaterra, 08193 Barcelona, Spain; joaquim.segales@irta.cat; 4 Wildlife Conservation Medicine Research Group (WildCoM), Departament de Medicina i Cirurgia, Facultat de Veterinària, Universitat Autònoma de Barcelona, 08193 Barcelona, Spain; carmeriutordfe@gmail.com; 5 Research and Conservation Department, Zoo de Barcelona, 08003 Barcelona, Spain; 6 UAB, Centre de Recerca en Sanitat Animal (CReSA, IRTA-UAB), Campus de la Universitat, Autònoma de Barcelona (UAB), 08193 Bellaterra, Spain; 7 Departament de Sanitat i Anatomia Animals, Facultat de Veterinària, Universitat Autònoma de Barcelona (UAB), 08193 Bellaterra, Spain

**Keywords:** molecular epidemiology, porcine circovirus 3 (PCV-3), Spain, wildlife species

## Abstract

Porcine circovirus 3 (PCV-3) is the third member of the family *Circoviridae*, genus *Circovirus*, able to infect swine. A high prevalence of viral DNA has been recorded in wild boars. Recently, PCV-3 DNA was identified in Italian wild ruminants. Based on these previous results, this study assessed the frequency of PCV-3 DNA detection in free-ranging ruminants and *Lagomorpha* species in Spain. In addition, the genetic characterization of the PCV-3 PCR-positive samples was performed. A total of 801 serum samples, including red deer (*Cervus elaphus, [CE]*; n = 108), roe deer (*Capreolus capreolus, [CC]*; n = 87), Pyrenean chamois (*Rupicapra pyrenaica, [RP]*; n = 133), Iberian ibex (*Capra pyrenaica, [CP]*; n = 92), mouflon (*Ovis aries, [OA]*; n = 91), fallow deer (*Dama dama, [DD]*; n = 104), European rabbit (*Oryctolagus cuniculus, [OC]*; n = 101), and European hare (*Lepus europaeus, [LE]*; n = 85) from Catalonia (northeast Spain) were tested by conventional polymerase chain reaction (PCR) and, when positive, sequenced. Overall, PCV-3 DNA was found in three out of 801 analyzed sera (0.37%) corresponding to one red deer (1/108, 0.9%), one mouflon (1/91, 1.1%), and one fallow deer (1/104, 0.96%). None of the samples collected from *Lagomorpha* species resulted PCR positive. The partial genome sequences detected in positive samples displayed high identity with some PCV-3 sequences detected in wild boars and domestic pigs (99.7% and 100%, respectively). In conclusion, the present study indicated that free-ranging ruminant and *Lagomorpha* species are not relevant in the epidemiology of PCV-3 in Spain.

## 1. Introduction

Porcine circoviruses (PCV) are small non-enveloped DNA viruses with icosahedral symmetry, containing a unique single-stranded circular genome of about 1.7–2.00 kb [1,2]. To date, four members of the genus *Circovirus* (family *Circoviridae*) have been detected in pigs. Porcine circovirus 1 (PCV-1) was the first identified one and considered non-pathogenic for swine [3]. Porcine circovirus 2 (PCV-2) is the most economically important family member as it can cause the so-called porcine circovirus diseases (PCVD) [4]. Porcine circovirus 3 (PCV-3) has been identified not only in pigs experiencing different pathological conditions, e.g., respiratory and reproductive problems, porcine dermatitis and nephropathy syndrome (PDNS), myocarditis, and congenital tremors, but also in clinically healthy pigs [5,6,7,8]. Finally, the recently described porcine circovirus 4 (PCV-4), so far only in China, was detected in animals with respiratory disease, diarrhea or PDNS-like lesions [9].

Similar to PCV-2 [10,11], PCV-3 is reported to be widely distributed in European wild boar populations without associated mortalities [12,13,14,15]. In addition, the PCV-3 genome has been confirmed in both wild ruminants and their related hematophagous ectoparasites (ticks) [13]. Thus, the study of the role of wildlife in the epidemiology of PCV-3 should be further addressed.

The main objectives of this work were to assess the prevalence of PCV-3 in free-ranging ruminant and *Lagomorpha* species in Spain based on a retrospective large-scale molecular survey, and carry out the genetic characterization of the positive samples.

## 2. Results

### 2.1. PCV-3 Detection

Three out of the 801 (0.37%) analyzed sera resulted PCV-3 positive by PCR. These three sera corresponded to one red deer (CE-615/17) (1/108, 0.9%), one mouflon (OA1-649/17) (1/91, 1.1%), and one fallow deer (DD1-847/18) (1/104, 0.96%). These three positive animals originated from the Lleida region. All the specimens collected from the rest of the wild ruminant and *Lagomorpha* species resulted negative for the presence of PCV-3 DNA.

### 2.2. Sequence and Genetic Characterization

**Figure 1:**
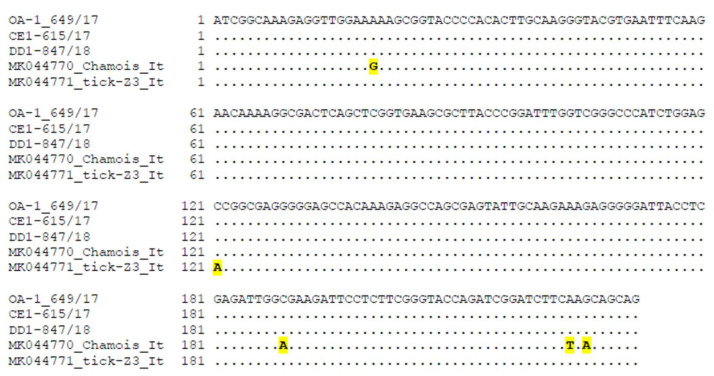
Alignment of the 232 nt overlapping replication sequences of the three porcine circoviruses 3 (PCV-3) obtained from wild ruminants in Spain with the available sequences from the PCV-3 detectedin chamois and tick in Italy. In yellow, nucleotide changes between the PCV-3 sequence detected in Italian chamois and tick when compared to the Spanish ones.

Sequencing of the complete genome was attempted but was not achieved, as only the capsid complete gene was obtained from the positive fallow deer and the partial replication gene was obtained from all three PCR PCV-3-positive wild ruminants. Failure to obtain the complete genome might be explained by the low amount of PCV-3 in serum samples. The alignment of the partial replication gene sequences showed a 100% identity between the sequence obtained from mouflon (OA1-649/17) with the sequences of one Spanish wild boar (MH751289 Spain WB 2017) and of domestic pigs from China (MG897477 China swine 2017) and Germany (MG014363 Germany swine 2015). This indicates that spillover infection cannot be discarded. Partial replication sequences of red deer (CE-615/17) and fallow deer (DD1-847/18) presented a 100% identity among them, and with sequences from domestic pigs from China (MH367846 China swine 2017) and the USA (KT869077 USA swine 2015). Replication nucleotide sequences from PCV-3 from the Italian ruminant were not included in the previous alignment since the analyzed regions were not totally coincident in both studies. Nevertheless, the similarity of the overlapping partial replication nucleotide sequence (232 nt) between the PCV-3 detected in Spanish wild animals and the virus detected in the Italian chamois (98.13%) and in the Italian tick (99.57%), indicated a certain difference among them (Figure 1). Capsid sequence alignment revealed a close relationship (pairwise distance = 0.006 and 0.008, respectively) between the sequences of the fallow deer (DD1-847/18) and the ones from Spanish wild boars (MH579737 Spain WB 2006 and MH579739 Spain WB 2010), suggesting a putative epidemiological link.

## 3. Discussion

According to previous studies, PCV-3 has been identified in different mammalian wild species such as wild boars and wild ruminants [12,13,14,15]. Interestingly, the frequency of the detection of viral DNA in wild boars originating from different European countries can be even higher (ranging from 23% and 50%) than the one reported in domestic pigs (from 6% to 32%) [2,7,8,12,13,14,15,16,17]. This high prevalence in wild boars was predictable, since wild boars and domestic pigs pertain to the same species, and most of the infectious agents infecting the domestic pigs are also circulating in the wild boars [18,19].

The current study confirmed the detection of PCV-3 DNA in different wild ruminant species, although at a very low prevalence. The obtained results are in line with the so-far only available study looking for the PCV-3 genome in wild species [13], where one out of nine chamois and two out of 50 roe deer were found PCV-3 PCR positive. Noteworthy, the Spanish wild ruminants’ samples originated from the same geographic area (Catalonia; northeast Spain) where high viral prevalence was previously reported in the Spanish wild boar population [14]. It may be speculated that contacts between wild boar and wild ruminant populations inhabiting the same geographic area could have favorable occasional spillover events between these species. Overall, however, it looks likely that the wild ruminants and *Lagomorphs* are not relevant in the epidemiology of PCV-3 in northeast Spain. 

## 4. Materials and Methods

### 4.1. Data and Specimen Collection

A total of 801 serum samples from red deer (CE; n = 108), roe deer (CC; n = 87), Pyrenean chamois (RP; n = 133), Iberian ibex (CC; n = 92), mouflon (OA; n = 91), fallow deer (DD; n = 104), European rabbit (OC; n = 101), and European hare (LE; n = 85) from Catalonia (NE-Spain) were analyzed for the presence of PCV-3 DNA. Wild ruminants were sampled randomly from their geographic areas of distribution. Thus, red deer, fallow deer, mouflon and Pyrenean chamois originated from Pyrenean and pre-Pyrenean counties (the Girona and Lleida regions, Catalonia, northeast Spain), the Iberian ibex were from Ports de Tortosa i Beseit National Game Reserve (the Tarragona region; Catalonia, northeast Spain). *Lagomorpha* species were randomly sampled in three different Catalan regions: Lleida, Girona, and Tarragona. All the samples were obtained from the heart or from the cavernous sinus of the dura mater from culled animals (during the hunting season from October to March) between 2015 and 2019 [20]. The gender and age of the hunted animals were registered. Centrifugation of blood samples was performed for 15 minutes at 1500 g. Derived sera were kept frozen (−20 °C) until analyses.

### 4.2. DNA Extraction, and PCV-3 Molecular Diagnosis

DNA extraction was performed from 200 µL of serum sample by using MagMAX™ Pathogen RNA/DNA kit (Applied Biosystem®). The presence of PCV-3 DNA was assessed by means of a conventional PCR assay detecting a 418-bp fragment of replication gene as previously described (Saporiti et al., 2019) [7]. Briefly, the 25 µL reaction volume contained 2.5 µL of extracted DNA, 12.5 µL of Go Taq®G2 Green Master Mix (Promega), 1 µL of each primer (10 µM), and 8 µL of sterile water. PCR was initiated by heating for five minutes at 94 °C, followed by 40 cycles of 1 min at 94 °C, 1 min at 60 °C, 1 min at 72 °C, and a final extension for 7 min at 72 °C. The PCR products were analyzed on a 1.5% agarose gel and stained with ethidium bromide.

### 4.3. PCV-3 Sequencing and Genetic Characterization 

For verification of PCV-3 PCR-positive samples, the 418-bp fragment of replication gene was sequenced. Sequencing PCR was conducted in a 50 µL reaction containing 3 µL of extracted DNA, 2 µL of each primer (10 µM), 10 µL of SuperFi Buffer (5X), 1 µL dNTP Mix (10 µM, Roche), 31.5 µL of sterile water, and 0.5 µL of Platinum SuperFi DNA polymerase (2 U/µL) (Invitrogen). The thermal protocol started with an initial heating at 95 °C for 2 min followed by 40 cycles of 95 °C for 30 s, 60 °C for 1 min and 72 °C for 1 min, followed by 7 min at 72 °C [7].

Attempts were made to obtain the complete genome of PCV-3 PCR assays using three specific pairs of primers (PCV-374–1144, PCV-31137–1561 and PCV-31427–433) reported by Fux et al. (2018) [21] with the slightly modified thermal conditions. The PCR reaction contained 2 µL of DNA template, 1 µL of each primer (10 µMl), 10 µL of SuperFi Buffer (5X), 2 µL of dNTP Mix (10 µM, Roche), 0.5 µL of Platinum SuperFi DNA polymerase (2 U/µL) (Invitrogen), and sterile water to bring the final volume up to 50 µL. The thermal protocol was as follows: activation of DNA polymerase at 98 °C for 5 min, 40 cycles of denaturation at 98 °C for 30 s, annealing at 55 °C for 1 min and extension at 72 °C for 2 min, followed by a final extension at 72 °C for 7 min. 

Purification of the amplicons was done using NucleoSpin® Gel and PCR Clean-up (Macherey-Nagel) following the manufacturer’s instructions. The quality and quantity of the genomic DNA were checked with BioDrop DUO (BioDrop Ltd, Cambridge, UK). The selected samples were sequenced by the Sanger method (BigDye® Terminator v3.1 Cycle Sequencing Kit) and analyzed using an ABI PRISM 3130xl Genetic Analyzer (Applied Biosystem®) at Servei de Genòmica, Universitat Autònoma de Barcelona (Spain). 

Partial replication and complete capsid nucleotide sequences were assembled by using ChromasPro v2.1.8 (Technelsyum). Partial replication sequences were aligned with 86 representative PCV-3 sequences using Clustal Omega (EMBL-EBI). The complete capsid sequence was aligned with capsid sequences available in the GenBank (n = 537). Pairwise distances were calculated using the Tamura–Nei model as the best fit model [22]. The sequences generated in the present work were submitted to the GenBank under the following accession numbers: MN901053 and MN954668–MN954670.
